# Mass spectrometry-based analysis of therapy-related changes in serum proteome patterns of patients with early-stage breast cancer

**DOI:** 10.1186/1479-5876-8-66

**Published:** 2010-07-11

**Authors:** Monika Pietrowska, Joanna Polanska, Lukasz Marczak, Katarzyna Behrendt, Elzbieta Nowicka, Maciej Stobiecki, Andrzej Polanski, Rafal Tarnawski, Piotr Widlak

**Affiliations:** 1 Maria Skłodowska-Curie Memorial Cancer Center and Institute of Oncology, Gliwice, Poland; 2 Silesian University of Technology, Gliwice, Poland; 3 Polish Academy of Science, Institute of Bioorganic Chemistry, Poznan, Poland; 4 Polish-Japanese Institute of Information Technology, Bytom, Poland

## Abstract

**Background:**

The proteomics approach termed proteome pattern analysis has been shown previously to have potential in the detection and classification of breast cancer. Here we aimed to identify changes in serum proteome patterns related to therapy of breast cancer patients.

**Methods:**

Blood samples were collected before the start of therapy, after the surgical resection of tumors and one year after the end of therapy in a group of 70 patients diagnosed at early stages of the disease. Patients were treated with surgery either independently (26) or in combination with neoadjuvant chemotherapy (5) or adjuvant radio/chemotherapy (39). The low-molecular-weight fraction of serum proteome was examined using MALDI-ToF mass spectrometry, and then changes in intensities of peptide ions registered in a mass range between 2,000 and 14,000 Da were identified and correlated with clinical data.

**Results:**

We found that surgical resection of tumors did not have an immediate effect on the mass profiles of the serum proteome. On the other hand, significant long-term effects were observed in serum proteome patterns one year after the end of basic treatment (we found that about 20 peptides exhibited significant changes in their abundances). Moreover, the significant differences were found primarily in the subgroup of patients treated with adjuvant therapy, but not in the subgroup subjected only to surgery. This suggests that the observed changes reflect overall responses of the patients to the toxic effects of adjuvant radio/chemotherapy. In line with this hypothesis we detected two serum peptides (registered m/z values 2,184 and 5,403 Da) whose changes correlated significantly with the type of treatment employed (their abundances decreased after adjuvant therapy, but increased in patients treated only with surgery). On the other hand, no significant correlation was found between changes in the abundance of any spectral component or clinical features of patients, including staging and grading of tumors.

**Conclusions:**

The study establishes a high potential of MALDI-ToF-based analyses for the detection of dynamic changes in the serum proteome related to therapy of breast cancer patients, which revealed the potential applicability of serum proteome patterns analyses in monitoring the toxicity of therapy.

## Background

Breast cancer is the most common malignancy in women and the fifth most common cause of cancer death (almost 1% of all deaths worldwide for both sexes counted) [1]. Breast cancer diagnosed at early clinical stages is relatively well cured (10-year disease-free survival usually exceeds 80%). Primary therapy for breast cancer is usually based on surgery, either radical or breast-conserving mastectomy. However, even in early stage cancer some patients are at high risk of metastasis or recurrence (usually about 20-30% of all patients), and they require adjuvant chemo- and/or radiotherapy. Because adjuvant treatment often has side effects, planning optimal therapy requires reliable prognostic and predictive markers of toxicity. Cancer markers currently used in clinical practice (e.g., staging and grading, proliferation capacity, receptor status) cannot determine exactly and undoubtedly which patients actually need adjuvant therapy. As a consequence, only a fraction of the patients who receive adjuvant chemo/radiotherapy will benefit from such treatment. This indicates a constant need for novel molecular markers for better prognosis and prediction of breast cancer therapy outcomes [2,3].

Proteomics, which is the study of the proteome - the complete description of the protein components of a cell or tissue, has shown increasing merit on cancer diagnostics in recent years. In contrast to the genome, the proteome is dynamic and its fluctuations depend on a combination of numerous internal and external factors. Identifying and understanding changes in the proteome related to disease development and therapy progression is the subject of clinical or disease proteomics [4,5]. Mass spectrometry-based analysis of the blood proteome is an emerging method of clinical proteomics and cancer diagnostics, and the low-molecular-weight (<15 kDa) component of the blood proteome is a promising source of previously undiscovered biomarkers [rev. in: [6-9]]. The proteomics approach that takes into consideration characteristic features of the whole proteome (e.g., mass spectra profiles) but does not rely on particular identified proteins, is called proteome pattern analysis or proteome profiling. In this approach multi-component sets of peptides/proteins (which are exemplified by ions registered at defined m/z values in the mass spectrum) define specific proteomic patterns (or profiles) that can be used for sample identification and classification [10-12]. Mass spectrometric methods particularly suitable for proteome pattern analysis are Matrix-Assisted Laser Desorption Ionization spectrometry (MALDI) and its derivative Surface-Enhanced Laser Desorption Ionization spectrometry (SELDI) coupled to a Time-of-Flight (ToF) analyzer. Numerous works have been published aiming to verify the applicability of MALDI- and SELDI-based analyses of the low-molecular-weight fraction of blood proteome for cancer diagnostics. Although no single peptide could be expected to be a reliable bio-marker in such an approach, multi-peptide profiles selected in numerical tests have been shown already in a few studies to have potential values for diagnostics of different types of cancer, though none of the identified peptide signatures has yet been approved for clinical practice [rev. in: [13-18]].

Several previous studies have addressed the possibility of applying mass spectrometry-based blood proteome pattern analysis in diagnostics of breast cancer. These works identified serum (or plasma) proteome patterns specific for patients with breast cancer at either early or late clinical stages [19-29]. Different methodological approaches, both experimental and computational, have been implemented in such studies, and the proposed proteome patterns (signatures) specific for breast cancer consisted of different peptide sets. However, several peptides that differentiated cancer and control samples appeared reproducibly when comparative analysis across different studies was performed [30,29]. This demonstrates the high potential of mass spectrometry-based analyses of the blood proteome pattern in diagnostics of breast cancer. A few previous studies have also used a mass spectrometry-based analysis of the blood proteome to address possible therapy-related changes or to identify prognostic/predictive factors. SELDI-ToF analysis identified one plasma peptide that was induced in the blood of breast cancer patients shortly after chemotherapy (most prominently after neoadjuvant therapy with paclitaxel), yet the presence of this peptide did not correlate with the outcome of therapy [31]. Similarly, increased levels of two peptides were observed shortly after infusion of docetaxel in the serum of breast cancer patients [32]. In addition, MS-based plasma proteome pattern analysis of post-operative blood samples disclosed peptides signatures that correlated with increased risk of metastatic relapse (the signature included haptoglobin alpha 1 chain, transferrin, C3a complement fraction, apolipoprotein C1 and apolipoprotein A1), which indicated possible prognostic value of such proteomics analysis [33].

In this work we aimed to identify the long-term changes in the serum proteome patterns that were related to therapy of early breast cancer patients.

## Methods

### Characteristics of patient groups

Seventy patients diagnosed with clinical stage I or II breast cancer were included in our study, of averaging 58 years of age (range 31-74 years). Patients were classified according to the TNM scale; the majority were scored as T1 and T2 (54% and 43%, respectively) as well as N0 and N1 (77% and 21%, respectively), and none had diagnosed metastases (all M0). All patients were subjected to either radical or conserving surgery to remove tumors (similar procedure of a general anesthetic was applied each time). The majority were subjected to adjuvant chemotherapy (9), radiotherapy (22) or chemo-radiotherapy (8), which was initiated 4-6 weeks following surgery (5 patients were treated with neoadjuvant chemotherapy before surgery). In addition, 54 patients showed increased expression of estrogen and/or progesterone receptors and were treated with a long-term anti-estrogen therapy. Blood samples from each patient were collected before the start of therapy (sample A) and 7-14 days after the surgery (sample B). A third sample (sample C) was collected either one year after the surgery or one year after the end of adjuvant radio/chemotherapy, which is termed "one year after the end of (basic) therapy" (this sample was usually collected 60-90 weeks after the corresponding sample A). The study was approved by the appropriate Ethics Committee (all participants provided informed consent indicating their voluntary participation) and was carried out at the Maria Sklodowska-Curie Memorial Cancer Center and Institute of Oncology, Gliwice Branch, between May 2006 and November 2009.

### Mass spectrometry analysis of serum samples

Blood samples (5 ml collected into Vacutainer Tubes, Becton Dickinson) were incubated for 30 min. at room temperature to allow clotting, and then centrifuged at 1000 g for 10 min. to remove clots. The sera were aliquoted and stored at -70°C. Samples were analyzed using an Autoflex MALDI-ToF mass spectrometer (Bruker Daltonics, Bremen, Germany); the analyzer worked in the linear mode and positive ions were recorded in the mass range between 2,000-14,000 Da. Mass calibration was performed after every four samples using appropriate standards in the range of 2.8 to 16.9 kDa (Protein Calibration Standard I; Bruker Daltonics). Prior to analysis each sample passed repeatedly 10 times through ZipTip C18 tip-microcolumns; columns were washed with water and then eluted with 1 μl of matrix solution (30 mg/ml sinapinic acid in 50% acetonitril and 0.1% TFA with addition of 1 mM n-octyl glucopyranoside) directly onto the 600 μm AnchorChip (Bruker Daltonics) plates. ZipTip extraction/loading was repeated twice for each sample and for each spot on the plate two spectra were acquired after 120 laser shots (i.e. four spectra were recorded for each sample). All samples were analyzed in a random sequence to avoid a possible batch effect.

### Data Processing and Statistical Analysis

The preprocessing of spectral data that included removing outliers by using Dixon test based on areas of the raw spectra, averaging of technical repeats, binning of neighboring points to reduce data complexity, removal of the spectral area below baseline and normalization of the total ion current (TIC), was performed according to procedures considering to be standard in the field [34,35]. In the second step the spectral components, which reflected [M+H]^+ ^peptide ions recorded at defined m/z values, were identified using decomposition of mass spectra into their Gaussian components as described elsewhere [29]. The average spectrum corresponding to samples A was decomposed into a sum of 400 Gaussian bell-shaped curves, by using a variant of the expectation maximization (EM) algorithm [36]. The model with 400 Gaussian components used in the current study was further post-processed with the aim to remove redundant components, which eventually led to obtaining Gaussian mixture decomposition with 334 not redundant components representing structures of the registered spectra. The Gaussian components were used to compute features of registered spectra (termed spectral components afterward) for all samples (A, B and C) by the operations of convolutions with Gaussian masks [29]. These spectral components were characterized by their abundances (or intensities), location along the m/z axis and standard deviation of corresponding Gaussian.

Comparisons between sets of spectra (A, B and C) were done separately for each of the spectral components. In order to estimate differences in intensities of spectral components between sets of samples, individual differential spectra were computed, paired with respect to time points (AB, AC and BC), and then one-sample t test was used with the null hypothesis that the mean values of intensities of the spectral components in the differential spectrum is equal to zero. Due to multiple spectral components analyzed, correction for multiple testing was necessary. Storey's q-values with thresholds for FDR (false discovery rate) equal to 0.05 were used to correct for multiple testing. The unsupervised clustering of spectral components based on their time courses was performed using the decomposition of three-dimensional probability density function into Gaussian components as described in [37]. To search for possible association between changes in abundances of spectral components and clinical parameters a method that we called "the modal analysis" was applied, aimed at identifying subgroups of patients with different patterns of changes in intensities of spectral components in time (between samples B and C). In this analysis the procedure of unsupervised clustering into two clusters was applied for each spectral component based on the K-means algorithm with the correlation function. Then the possible coincidence of the obtained clusters with subgroups defined by clinical parameters were assessed by using the chi-square test (with Yates correction) in the case of discrete-type parameters or the Kruskal-Wallis ANOVA test in the case of continuous-type parameters.

## Results

**Figure 1:**
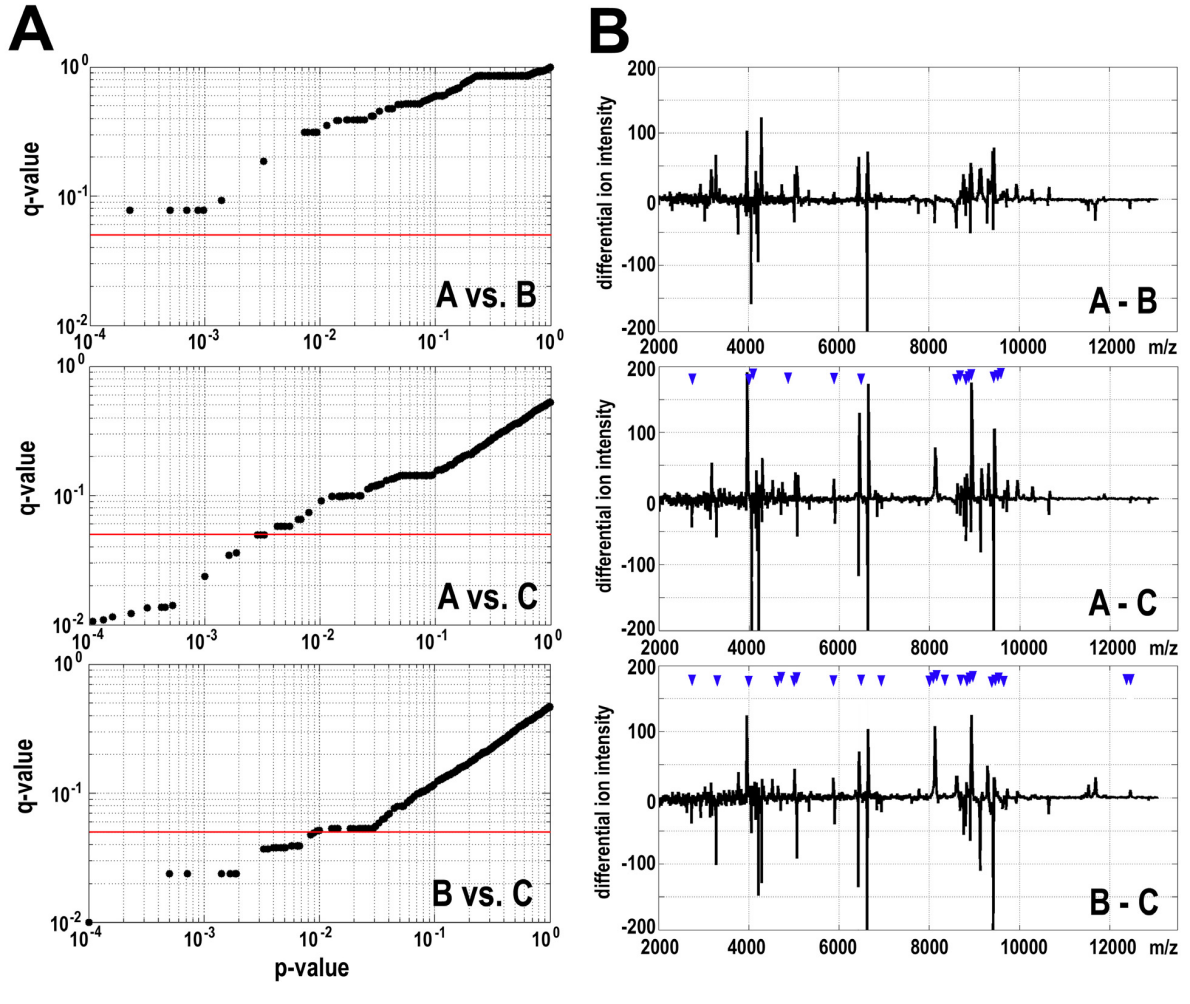
**Assessment of differences of proteome patterns specific for serum samples collected at different time points**. **A **- The q-values were plotted against the p-values of differences between compared samples A. B and C; each dot represents one spectral component, the red horizontal line represents a q-value cut-off equal to 0.05. **B **- Average differential spectra computed for each pair-wise comparison; blue arrowheads marked positions of spectral components that differentiated samples at high levels of significance (q-value < 0.05).

In the first step of analysis three pair-wise comparisons of mass spectra registered with MALDI-ToF system for samples collected before the start of therapy (sample A), after the surgical removal of tumor (samples B), and one year after the end of basic therapy (samples C) were performed for each patient to obtain individual differential spectra, and then the average differential spectra that described analyzed group of 70 patients were computed. For each of all spectral components (i.e. registered peptide ions) the significance of a difference in abundance between compared time points was characterized by its p-value and q-value; the latter one reflected significance of differences adjusted for multiple testing using the False Discovery Rate (FDR) approach. Figure 1A shows q-values plotted against p-values of such differences for each spectral component in three pair-wise analyses; a q-value equal to 0.05 was chosen here as the rigid significance cut-off level. We did not find significant differences between serum samples collected before the start of therapy and after surgery (A vs. B). In marked contrast, several spectral components showed significant changes in their abundance when we compared samples collected before the start of therapy and one year after the end of therapy (A vs. C), as well as samples collected after the surgery and one year after the end of therapy (B vs. C). Figure 1B shows location of such differentiating components marked along corresponding average differential spectra. Fourteen spectral components changed their abundance significantly between samples A and C, while 24 spectral components changed their abundance significantly between samples B and C. Importantly, the same 8 spectral components differentiated samples C from both samples A and samples B (approximate registered m/z values = 2742, 3992, 5877, 6489, 8888, 8931, 8942 and 8973 Da). When a less rigid significance cut-off level q-value equal 0.1 was considered 69 spectral components appeared to differentiate samples B and C, while only 6 spectral components differentiated samples A and B (Figure 1A). The m/z values of registered spectral components were annotated at the knowledge base EPO-KB (Empirical Proteomic Ontology Knowledge Base) [38] aiming at hypothetical identification of serum peptides (assuming their mono-protonation and allowing for a 0.5% mass accuracy limit). Such analysis allowed hypothetical annotation of 22 out of 69 components that differentiated samples B and C. Table 1 shows examples of spectral components that differentiated samples B and C. We conclude that serum proteome patterns were similar when samples collected before the start of therapy and after the surgery were compared. In marked contrast, proteome patterns of serum samples collected one year after the end of basic therapy changed when compared to both types of samples collected at earlier time points.

**Table 1:** Examples of spectral components that differentiated serum samples collected after surgery and one year after the end of basic therapy.

			q-value of the difference		
			
Component m/z	Hypothetical identity	Δ	All patients	Adjuvant therapy	Only surgery
**2,742**			0.050	0.118	0.275

**3,263**	FGA (fr.576-604)	I	0.053	0.084	0.506

**3,302**	-	I	0.039	0.238	0.150

**3,992**	-	D	0.039	0.136	0.182

**4,038**	-	I	0.099	0.099	0.510

**4,524**	APP (fr.672-713)	D	0.053	0.084	0.462

**4,642**	-	D	0.049	0.201	0.157

**4,713**	-	I	0.038	0.199	0.152

**5,048**	-	I	0.025	0.081	0.262

**5,067**	-	I	0.038	0.084	0.367

**5,377**	-	D	0.053	0.016	0.344

**5,403**	-	D	0.159	0.016	0.181

**5,877**	FGA (fr.581-633)	D	0.039	0.084	0.238

**5,998**	-	D	0.076	0.084	0.344

**6,489**	-	D	0.038	0.084	0.272

**6,939**	TTR (fr.21-147)	I	0.001	0.016	0.119

**8,032**	-	D	0.037	0.090	0.193

**8,095**	-	D	0.038	0.128	0.137

**8,156**	-	D	0.039	0.201	0.150

**8,354**	C3 (fr.672-739)	D	0.038	0.238	0.101

**8,844**	-	D	0.053	0.086	0.251

**8,888**	VTN (fr.402-478)	D	0.039	0.206	0.152

**8,931**	C3 (fr.672-747)	D	0.039	0.238	0.106

**8,942**	APOA2 (fr.24-100)	D	0.024	0.084	0.204

**8,973**	-	D	0.024	0.099	0.193

**9,419**	APOC3 (fr.21-99)	I	0.024	0.084	0.207

**9,519**	-	D	0.039	0.207	0.150

**9,990**	-	D	0.099	0.081	0.333

**12,359**	-	D	0.038	0.238	0.151

**12,453**	-	D	0.048	0.154	0.244

Shown are: approximate m/z value, hypothetical identity, average change in abundance between samples B and C (D - decrease, I - increase) and q-value of the difference (the Storey test). Components that differentiated samples B and C both in group of all patients and patients subjected to adjuvant therapy are underlined.

**Figure 2:**
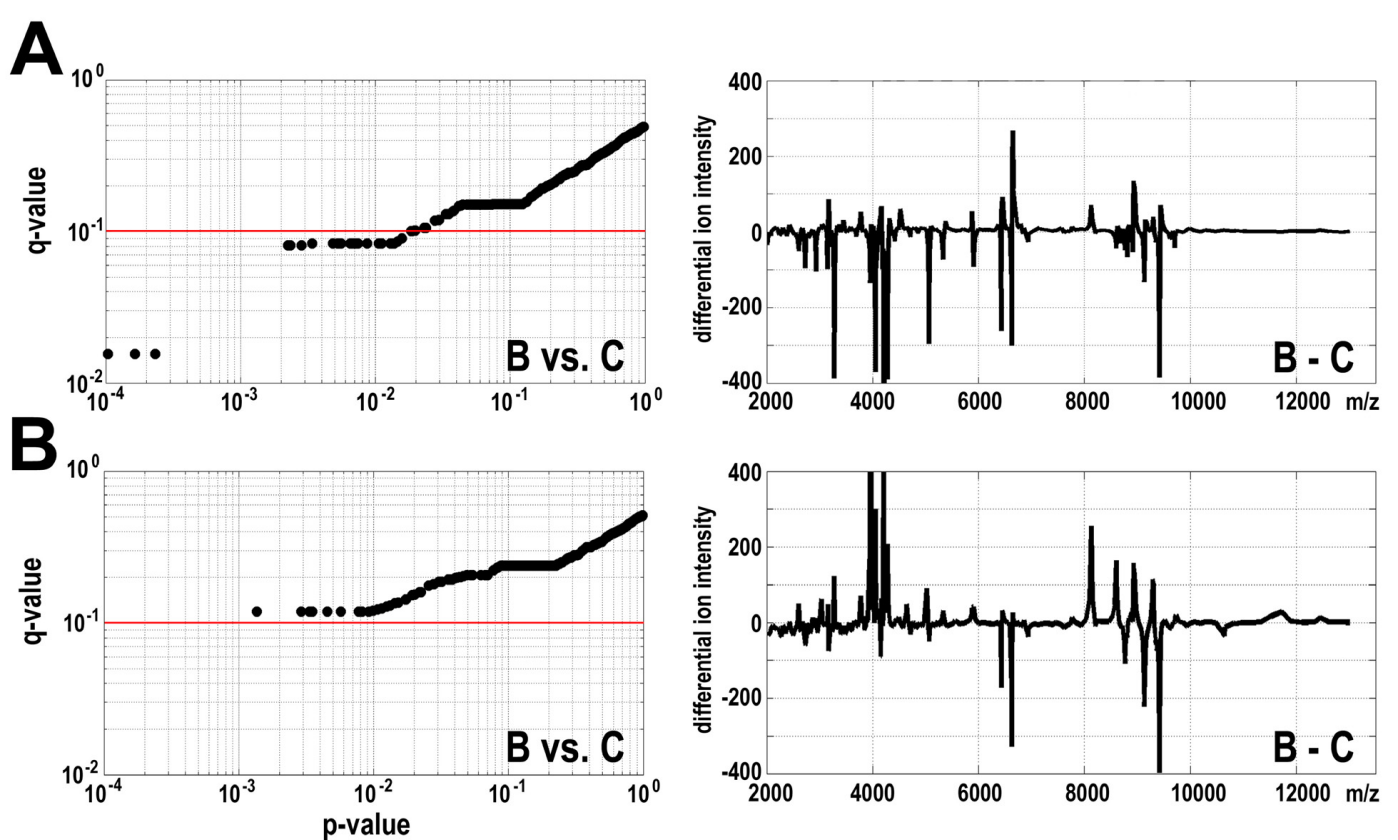
**Changes in serum proteome patterns specific for subgroups of patients**. **A **- Analysis of the group of patients subjected to surgery and adjuvant therapy. **B **- Analysis of the group of patients subjected only to surgery. **Left **- the q-values are plotted against the p-values of differences between samples B and C; each dot represents one spectral component, the red horizontal line represents a q-value cut-off equal to 0.1. **Right **- average differential spectra for samples B and C.

In order to test the hypothesis that observed differences were related to adjuvant radio/chemotherapy two subgroups of patients were analyzed in parallel: patients subjected only to surgery (26 persons) and patients treated with adjuvant therapy (39 persons). As expected, in neither subgroup significant differences between samples A and B were found. Surprisingly, also when samples A and C were compared differences for none of spectral components reached the level of statistical significance (q < 0.1) in both groups of patients, which apparently was related to smaller numbers of samples in these subgroups. However, clear differences were observed between two groups of patients when samples B and C were compared. Several spectral components changed their abundance significantly between these two time points when samples from patients subjected to adjuvant therapy were analyzed. The q-value of the difference in abundance of 26 spectral components reached the level of <0.1 when serum samples from this subgroup were analyzed (Figure 2A). In marked contrast, none of spectral components changed their abundance significantly between time points B and C when samples of patients subjected only to surgery were analyzed (Figure 2B). Noteworthy, 16 out of 26 spectral components that differentiated samples B and C in the subgroup subjected to adjuvant therapy also differentiated samples B and C when the group of whole patients were analyzed (at the level of q-value < 0.1; Table 1). We conclude that differences in serum proteome patterns observed between samples collected after the surgery and one year after the end of basic therapy were specific for the group of patients subjected to adjuvant therapy, and this reflects changes related to this treatment.

**Figure 3:**
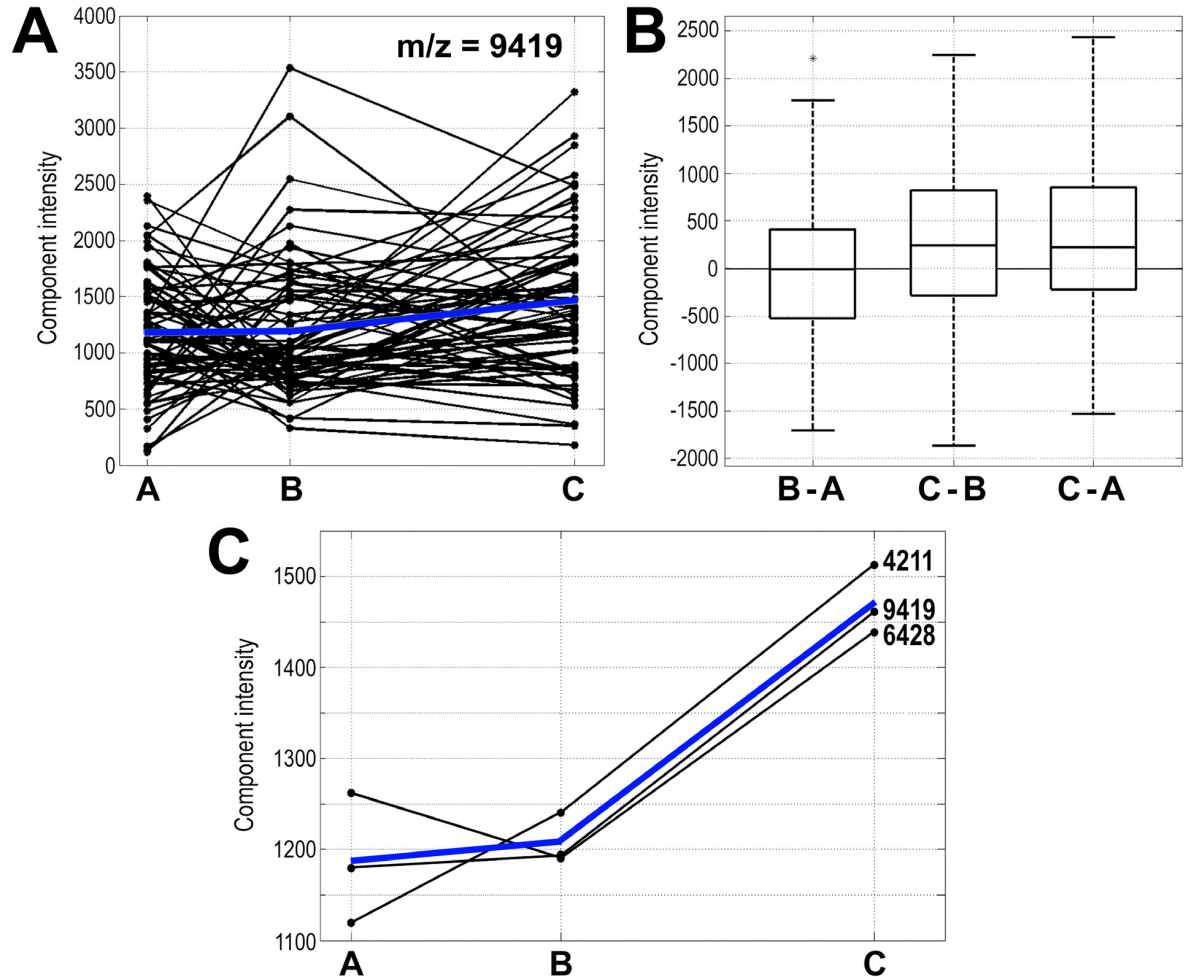
**Example of time course-related changes in the abundances of spectral components**. **A **- Individual time courses of changes in the abundance of spectral components registered at the approximate m/z value 9419 Da in samples collected from 70 patients at three time points (dots connected with black lines); blue lines represent the average for all patients. **B **- Box-plots represent quantification of differences in the abundance of the 9419 Da spectral component in samples collected for each of 70 patients between three time points; shown are minimum, lower quartile, median, upper quartile, maximum values and outlier marked with asterisk (q-values of the significance of differences were 0.856, 0.024 and 0.065 for B-A, C-B and C-A, respectively). **C **- Cluster that contained spectral components registered at approximate m/z values 4211, 6428 and 9419 Da. For each of three components shown are average intensities for samples collected from 70 patients at different time points (dots connected with black lines), the blue line represents averages for all components; the cluster represents [A≥B<C] type.

Based on the abundance of each spectral component registered in serum samples collected at different time points for each patient, individual "time courses" were established. Then, average time courses were computed for each spectral component, which characterized its general behavior in samples from a group of patients. Such average time courses were used in cluster analysis to extract spectral components whose abundance in samples changed in a specific way. We separated 30 clusters, which number described the dataset optimally according to Bayesian information criterion [39] (not shown). Figure 3 shows an example of individual time courses of changes in abundance of the spectral component registered at approximate m/z value 9419 Da (putatively fragment of apolipoprotein C3), which differentiated samples B and C, and the 3-element cluster that contained this particular component. The cluster analysis was performed for the whole group of patients (n = 70) and the group of patients subjected to adjuvant therapy (n = 39); characteristics of identified clusters are shown in Table 2. As expected, the majority of spectral components belonged to a few clusters where the average abundance of components did not change significantly between consecutive time points (i.e., t-test p-value > 0.05 or average abundance changed for less than 5% in clusters with a few components). Such [A = B = C] type of clusters contained 78% and 63% of the spectral components when the group of all patients and patients subjected to adjuvant therapy were analyzed, respectively. Average abundance of several spectral components increased between samples collected after surgery (samples B) and one year after the end of therapy (samples C); these components formed [A<B<C] or [A≥B<C] types of clusters. These types of clusters consisted of 16% and 25% of the components for the group of all patients and patients subjected to adjuvant therapy, respectively. Fewer spectral components decreased their average abundance between samples B and C. These formed [A>B>C] or [A≤B>C] types of clusters, which consisted of 5% and 3% of the components for the group of all patients and patients subjected to adjuvant therapy, respectively. In line with data presented on Figure 1, the minority of spectral components changed their abundance between samples A and B but not between samples B and C, and belonged to [A ≠ B = C] types of clusters. These data showed that a substantial number of spectral components changed their abundance when analyzed in consecutive samples collected after surgery and one year after the end of therapy, and confirmed that such time-related changes are expressed predominantly in group of patients subjected to adjuvant therapy.

**Table 2:** Characteristics of clusters of spectral components that behave similarly in samples collected at analyzed time points.

Type of cluster	All patients (n = 70)		Adjuvant therapy (n = 39)	
	
	Number of clusters	Number of components	Number of clusters	Number of components
A = B = C	7	261	6	212

A < B < C	4	4	7	9

A ≥ B < C	12	49	10	74

A ≤ B > C	2	7	3	11

A > B > C	2	9	0	0

A ≠ B = C	3	4	4	28

**Figure 4:**
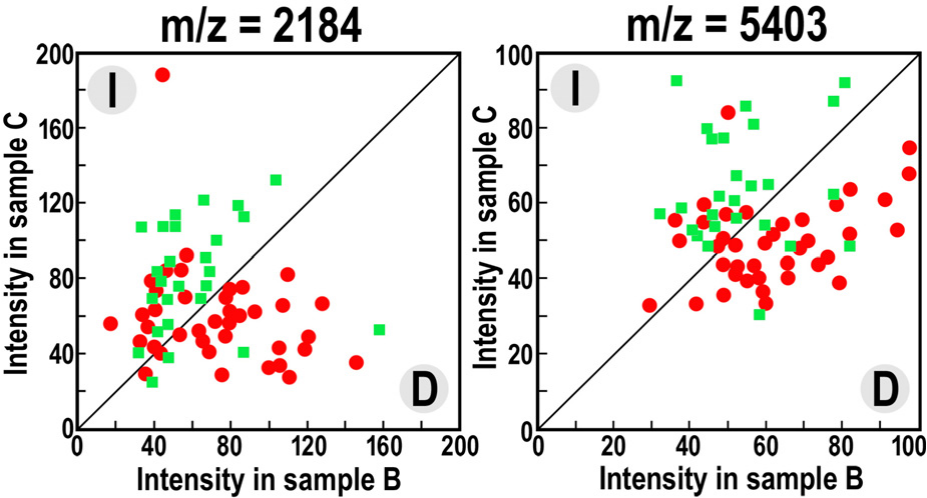
**Changes in the abundance of spectral components that correlates with the type of therapy**. Shown are intensities of two spectral components, m/z = 2184 and 5403, in serum samples collected after surgery (sample B) and one year after the end of basic therapy (sample C). Each component either increased (I) or decreased (D) between samples B and C (upper left and bottom right halves of the graph, respectively); the red dots represent patients subjected to surgery and adjuvant therapy, green boxes represent patients subjected only to surgery.

In the next step we analyzed whether changes in abundance of a given spectral component registered in samples collected after surgery and one year after the end of basic therapy correlated with clinical data; two clusters of samples were separated where the component's intensity either increased or decreased between points B and C. We found that modality in changes of two spectral components (m/z = 5403 and 2184 Da) correlated significantly with the scheme of therapy (p-value 0.00003 and 0.00005, respectively). Figure 4 shows that the abundance of both components most likely decreased in serum of patients treated with the adjuvant therapy while these increased in serum of patients subjected only to surgery. In addition, we analyzed the possible associations between modality in changes of each spectral component and each of 20 available "classical" clinical features, which among others included: age, different measures of staging and grading, estrogen and progesterone receptor expression, HER2 status, leukocyte and hemoglobin levels. Importantly, a correlation between any of these clinical features and changes in intensity of any spectral component did not remain statistically significant when a Bonferroni correction for multiple testing was applied. Noteworthy, however, among ~200 pairs of features (i.e., spectral component vs. clinical feature; 8000 pairs were possible overall) that showed some tendency to associate (i.e. uncorrected p-value < 0.05), there were 43 spectral components that correlated with expression of either the progesterone or estrogen receptor. This tendency suggests that certain changes observed between samples collected after surgery and one year after the end of basic therapy were related to anti-estrogen treatment ongoing in patients with a high level of expression of estrogen/progesterone receptors.

## Discussion

We had previously implemented the Gaussian mixture model to decompose MALDI spectra of the low-molecular-weight fraction of the serum proteome for untreated patients diagnosed with early stages of breast cancer and corresponding healthy controls to identify and quantify spectral components that corresponded to peptides registered as specific [M+H]^+ ^molecular ions. This approach allowed us to identify spectral components (corresponding to serum peptides) whose abundance was different between groups of patients and healthy donors, and then such differentiating components were used to build a multi-component cancer classifier [29]. The strategy for construction of such classifiers involves comparison between spectral features (i.e., abundances of particular components) specific for analyzed groups (e.g., comparing average spectra for patients and controls). Here we aimed to analyze dynamic changes in proteome patterns specific for each individual patient, which required a different methodological approach. The first step in this approach was to compare spectra registered for serum samples taken from the same donor at three different time points of therapy (i.e., before the start of therapy, after the surgical removal of the tumors, and one year after the end of basic therapy) that allowed obtaining individual differential spectra. Based on the individual differential spectra, the average differential spectra were computed to identify spectral components (i.e. peptide molecular ions) that differentiated the analyzed time points in general.

We found that registered mass profiles (proteome patterns) were similar when serum samples were collected before the start of therapy and after the surgery, which indicated that resection of the tumor did not have an immediate influence upon the serum proteome of patients. However, clear differences between serum samples collected at either of these "early" time points and serum samples collected one year after the end of basic therapy were identified. Among registered peptide ions that changed their abundances and were hypothetically annotated at the proteomic knowledge base EPO-KB [38] were fragments of apolipoprotein A2 (APOA2), apolipoprotein C1 (APOC1), apolipoprotein C2 (APOC2), apolipoprotein C3 (APOC3), amyloid beta A4 (APP), complement C3 (C3), c-c motif chemokine 13 (CCL13), cystatin-3 (CST3), neutrofil defensin-3 (DEFA), fibrynogen alfa chain (FGA), haptoglobin (HP), inter-alpha-trypsin inhibitor heavy chain H4 (ITIH4), platelet factor 4 (PF4), transthyrein (TTR), neurosecretory protein VGF (VGF) and vitronectin (VTN). Noteworthy, these serum proteins were previously reported to be related to breast cancer [25,30,33].

It is noteworthy that the most significant changes in proteome patterns were observed in serum samples collected one year after the end of adjuvant radio/chemotherapy. There was no significant correlation identified between features of tumors (e.g., its clinical staging and grading) and changes in the abundance of specific components of the serum proteome (previously we showed similar serum proteome profiles for patients with different clinical staging of the disease, i.e. T1 vs. T2, N0 vs. N1 and G1/2 vs. G3 [29]). In contrast, there were two peptides identified (namely spectral components registered at m/z 2184 and 5403 Da) whose changes in abundance correlated with the type of treatment (i.e., their intensities decreased after adjuvant therapy while increased in patients treated only with surgery). In addition, certain differences in serum proteome patterns were observed among patients differing in expression of progesterone/estrogen receptors, which most apparently corresponded to ongoing anti-estrogen treatment of patients with high expression of these receptors. Moreover, similarity between mass profiles characteristic for serum samples collected one year after the end of therapy and serum samples collected from healthy persons was not higher than similarity between serum samples collected from breast cancer patients before the start of therapy and samples of healthy controls (data not shown). All this suggests collectively that changes in the proteome pattern observed one year after the end of basic therapy (either surgery alone or adjuvant treatment) reflects a long-term response of patients' organs to the toxic effects of adjuvant radio/chemotherapy rather than a "curation" of the tumors. In the time frame of our study, tumor recurrence or metastasis was diagnosed only in four woman, thus finding a correlation between specific features of serum proteome patterns and the effectiveness of therapy is not possible at this early stage of our investigation.

Only a few publications have addressed the question of detecting therapy-related changes in the mass profiles registered for blood samples collected from breast cancer patients. SELDI-ToF analysis of the plasma proteome of breast cancer patients who underwent paclitaxel-based neoadjuvant treatment revealed one peptide (m/z = 2790 Da), which specifically increased in its abundance [31]. Similar analysis of the serum proteome of patients infused with docetaxel revealed two peptides (m/z = 7790 and 9285 Da), which changed their abundances in response to the treatment [32]. However, these taxane-induced changes were detected in samples collected just few days (or hours) after the treatment. There is only one small-scale study that has addressed the long-term effects related to the treatment of breast cancer patients. In this pilot study [20], 16 paired serum samples collected from breast cancer patients before the treatment and post-treatment (6-12 months after surgery and at least one month after the end of adjuvant therapy) were analyzed using SELDI-ToF; the treatment scheme was heterogenous in this group and based on surgery alone, or surgery supplemented with neoadjuvant chemotherapy or adjuvant chemo/radiotherapy. It was found that three peptides (m/z = 2276, 4892 and 6194 Da) increased their abundance in serum collected post-treatment. Noteworthy, both pre-treatment and post-treatment samples retained specific features of mass profiles that differentiated them from serum samples collected from healthy donors [20]. Results of that pilot study are indeed in agreement with our findings, which both indicate that changes in serum proteome patterns observed after long-term treatment reflect responses of patients to therapy but not restoration of the "normal healthy" pattern of the serum profile.

## Conclusions

Here we established the high potential of MALDI-ToF-based analyses for detection of dynamic changes in serum proteome mass profiles that result from therapy of breast cancer patients. We found that surgical resection of tumors did not have an immediate effect on the serum proteome. On the other hand, significant long-term effects were observed in the serum proteome one year after the end of basic treatment. We believe that the observed changes reflect overall responses of the patients to the toxic effects of adjuvant radio/chemotherapy. Our results reveal the potential applicability of mass spectrometry-based serum proteome pattern analyses in monitoring the toxicity of therapy.

## Competing interests

The authors declare that they have no competing interests.

## Authors' contributions

MP - performed experiments, interpreted results, JP - performed mathematical modeling and statistical analyses, LM - performed experiments, interpreted results, KB - collected and interpreted clinical data, EN - collected and interpreted clinical data, MS - designed and interpreted MS data, drafted manuscript, AP - designed mathematical modeling, drafted manuscript, RT - designed and interpreted clinical part of the study, drafted manuscript, PW - designed and interpreted experiment, prepared final manuscript. All authors read and approved the final manuscript.
